# Reading Independently and Reading With a Narrator: Eye Movement Patterns of Children With Different Receptive Vocabularies

**DOI:** 10.3389/fpsyg.2018.01753

**Published:** 2018-09-24

**Authors:** Zhuqing Su, Yifang Wang, Yadong Sun, Jinhong Ding, Zhuoya Ma

**Affiliations:** ^1^Department of Psychology, Capital Normal University, Beijing, China; ^2^Department of Preschool Education, Yichun Early Childhood Teachers College, Yichun, China

**Keywords:** children, picture books, eye movement, reading styles, receptive vocabulary

## Abstract

This study examined the effects of two reading styles (i.e., reading with a narrator and reading independently), receptive vocabulary and literacy on children’s eye movement patterns. The sample included 46 Chinese children (aged 4–6 years) who were randomly assigned to two reading styles and read the same picture book on a screen. The results indicated that the higher the children’s receptive vocabulary was, the sooner they fixated on the text. Overall, the children’s fixation probability (i.e., the time spent viewing the text zones as a proportion of full-page viewing time during each period) decreased with time when reading independently but increased with time when reading with a narrator. For children in senior kindergarten, reading with a narrator is thought to help establish and consolidate the links between speech and text and thus promote reading acquisition.

## Introduction

Substantial importance has been attached to improving the quantity and quality of reading among citizens in China, and *Reading for All* has twice been included in the “Report on the Work of the Government" (Qian, 2005). Reading cannot only increase people’s knowledge and enrich their spiritual lives but can also improve the quality of the national culture. Hence, the government promotes reading for all, and forms of reading are becoming more diversified and modernized. Preschool is a critical period for cultivating children’s reading habits, and numerous studies have demonstrated that reading during early childhood has a positive impact on children’s language skills (Felton, 1992; Evans and Saint-Aubin, 2013), emotional intelligence (Doyle and Bramwell, 2006; Nikolajeva, 2013) and other aspects of development. Picture books combine two types of symbols, namely, pictures and short sections of text (which, in this paper, refers to Chinese characters), to tell a story. Such books are intuitive, easy to understand and interesting. Thus, children’s early reading activities involving picture books have attracted increasing attention from children and parents.

Various reading styles may be employed when reading picture books. Sharolyn et al. (2016) described shared book reading and interactive reading, two terms that refer to adults’ encouragement of children’s active participation when they read books together. When employing these reading styles, adults ask children open-ended questions about the book and expand on their feedback to help children learn to express themselves more accurately. Evans and Saint-Aubin (2013) discussed another “adults reading to children” style that does not involve adult–child interaction, in contrast to shared book reading and interactive reading. In this process, adults read to children in a straightforward manner without pointing, emphasizing, or explaining. With the development of multimedia technology, printed books are no longer the only form of reading: children can read books on electronic devices so that they can read not only by “looking” with their eyes but also by “listening” with their ears, which substantially increases interest in reading among children who are initially unenthusiastic (Maynard and McKnight, 2001; Maynard, 2010). In this study, we simulated an electronic reading situation for children and defined two reading styles; for both styles, the picture books were presented on a computer screen, and the children were allowed to proceed at their own pace. For the first reading style, the participants could listen to a recording of the text after they turned each page by clicking with a mouse; for the second reading style, there was no recording that matched the text after each page was turned. There were no other differences between the two styles. We defined the first reading style as reading with a narrator and the second as reading independently.

Studies of picture book reading have shown that children pay very little attention to text, regardless of whether they are reading independently (Evans et al., 2009) or reading with a narrator (Evans and Saint-Aubin, 2005; Justice et al., 2005). Unlike reading independently, reading with a narrator includes sound information. Therefore, we wondered whether the added auditory information affects the amounts of time that children allocate to pictures versus text when reading picture books. Han et al. (2011) directly compared the characteristics of children’s eye movement for these two reading styles and found that the time spent on pictures did not differ. For the text zones, however, the viewing time of the children (aged 5–6 years) who were reading independently was longer than that of the children who were reading with a narrator. According to Nikolajeva and Scott (2006), picture book reading is a process that involves matching text with pictures, and each component promotes understanding. When reading with a narrator, sound information is added, and the connection between the sound and the printed word is very direct (i.e., children can frequently pronounce a word while failing to grasp its meaning) (Siegel, 1983). Therefore, listening to adults provides a scaffold for children with limited reading ability to explore a story by simultaneously listening to auditory information and looking at pictures (Evans and Saint-Aubin, 2005; Martin-Chang and Gould, 2012). For that reason, children pay less attention to text when reading with a narrator than when reading independently.

However, children do not show an overwhelming preference for the pictures all the time. A longitudinal study of children’s reading activities concluded that children experience a process of “from pictures to text” (Sulzby, 1985). Specifically, children focus primarily on pictures when they begin to read, gradually focus on both pictures and text, and eventually actively focus on text and become mature readers. This developmental process is also a process of gradual improvement in reading skills. Similarly, Roy-Charland et al. (2007) found that with the advancement of grade level and reading skill (in their study, reading skill was considered to co-vary with grade level, at least to an extent), the duration of text viewing and fixation on text gradually increased. Roy-Charland et al. (2007) used the Stroop effect to explain these findings. In classical Stroop tasks (1935), it takes less time to name an ink color when the ink color is consistent with the meaning of a word (i.e., when the word “blue” is written in blue ink) than when the ink color conflicts with the meaning of the word (i.e., when the word “blue” is written in red ink), which suggests that the connection between a printed word and its meaning is more direct than the connection between a printed word and its color. This effect is thought to prompt children to automatically focus on text when they look at it. Moreover, once children form the connection between printed words and their meanings, the Stroop effect becomes very strong (Everatt et al., 1999; Wright and Wanley, 2003; but see Jerger et al., 1993). However, this connection usually forms at approximately 7 years of age (Comalli et al., 1962). Children in lower grades (i.e., kindergarten), whose reading skills have not developed as fully, are unable to connect printed words and their meaning automatically. Thus, they primarily attend to pictures.

Picture book reading is not only a process of “from pictures to text,” which reflects the development of children’s reading skills; it is also a process of receiving information from the external environment. Children’s receptive vocabulary and literacy are strong predictors of their reading ability (Coates and Lewis, 1984; Evans et al., 2009). Evans et al. (2009) tracked the eye movements of children aged 59–71 months while they were reading a simple, clear alphabet book (each page featured a single large uppercase letter, one prominent word in uppercase, a single corresponding object, and the same bear). The results suggested that children with a larger receptive vocabulary more quickly fixated on letters and words. Both vocabulary and letter knowledge accounted for 23–56% of the variance in the seven dependent variables. Similar results were reported by Evans and Saint-Aubin (2013), who analyzed the vocabulary acquisition of 36 children aged 50–62 months after repeated reading of a storybook (7 days). The results of their study revealed that the children’s fixation on the text was significantly correlated with their letter knowledge. Thus, children’s receptive vocabulary and letter knowledge are important influences on the reading process and warrant further study.

The eye-tracker, which can record eye movements in real time, has become an important tool for investigating reading behavior. Previous research on preschool children’s picture book reading activities, i.e., comparing eye movement patterns either among different reading styles or among children with different receptive vocabulary sizes, have often focused statically on different interest zones on the whole-page. However, we do not think that the static analysis method is sufficient. According to Nikolajeva and Scott (2006), picture book reading is a process that involves matching text with pictures. To determine whether this is true, we must examine children’s reading process in depth. In addition, in our practice, we have also found that children’s distribution of text and picture reading time is different at different stages of reading a book. Therefore, we chose to subdivide the children’s whole-page reading time to examine the changes in eye movement patterns over the time course. Specifically, we divided each page into 10 viewing periods, i.e., the duration from first fixation to last fixation. For each period, the time spent viewing the text zones that accounted for the entire page was calculated, and eye movement trends throughout the process of reading a picture book were examined. We thus viewed picture book reading as a continuous process; this dynamic analysis method may be a more accurate and detailed way to investigate the topic. The picture book used in this study was clear and simple with an apple tree and a protagonist (a little mouse) appearing on every page. Thus, we defined the protagonist as one of two important zones of interest (the other being the text zone).

The first purpose of this study was to determine whether there are differences in eye movement patterns when children read picture books independently versus when they read them with a narrator. Considerable research has shown that children primarily look at pictures when reading picture books (Justice et al., 2005; Evans and Saint-Aubin, 2005, 2013; Evans et al., 2009). Therefore, regardless of which reading style is assigned, it might be expected that children will show an overwhelming preference for the protagonist and that there will be no difference in the time spent viewing the protagonist between the two reading styles. Given the special method of subdividing the entire viewing time into smaller periods, we propose no *a priori* hypothesis about children’s reading patterns in the text zone.

A second purpose was to examine the influence of Peabody Picture Vocabulary Test (PPVT) scores and literacy skills on children’s eye movement patterns while reading a picture book. According to Coates and Lewis (1984), children’s receptive vocabulary and literacy skills are strong predictors of their reading ability. Consequently, we hypothesized that the influence of these two variables on reading patterns would mainly affect the text zone; in other words, the higher the children’s PPVT scores and literacy levels were, the sooner and the longer they would fixate on the text. Through this research, we hope to deepen the understanding of the characteristics of early childhood reading in order to provide a reference for parents who wish to determine the appropriate reading style for their children.

## Materials and Methods

### Participants

The participants included 54 children from a kindergarten in Beijing. All the children were native Chinese speakers with no history of gross motor, hearing, vision or language problems. The children were randomly assigned to two reading groups. In the formal experiment, eight children performed successive mouse clicks or made large body movements, so their eye movement data were invalid. The final sample consisted of 46 children, with 23 children in each group; 15 boys and 8 girls, ranging in age from 55 to 76 months, were assigned to the independent-reading group, and 12 boys and 11 girls, ranging in age from 54 to 78 months, were assigned to the reading-with-a-narrator group. See details in **Table 1**.

**Table 1:** Descriptive statistics of the children.

Reading style	*N*	Age	Gender (boy/girl)	PPVT scores	Literacy
Independent	23	67.14 (5.49)	15/8	69.96 (19.73)	14.35 (9.01)
Narrator	23	69.18 (5.87)	12/11	71.91 (18.76)	16 (7.82)

*PPVT refers to the Peabody Picture Vocabulary Test. “Independent” means independent reading, and “narrator” means reading with a narrator*.

### Materials

**FIGURE 1:**
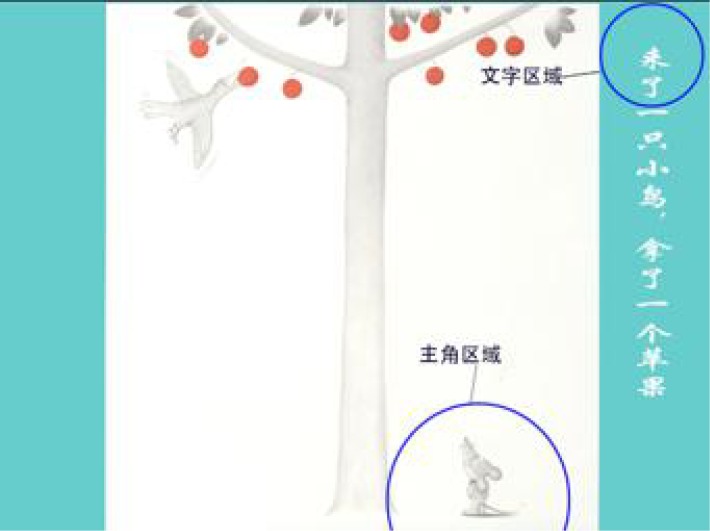
Sample page of the picture book used in the experiment. The English translation of the sentence in the Chinese book is “A bird comes and takes an apple.”

A picture book entitled “The Little Mouse Wants Apples” (Zhongjiang and Shangye, 2014) was used. The book tells a simple and interesting story about a small mouse who eats apples with the help of friends. Six types of animals are mentioned in turn, except the little mouse. We confirmed that all the children knew these animals and that they had not previously read the book. The book is aimed at children aged 3–6 years. Three pages of the book were excluded, and 13 pages were presented to the children. The first page (home) was the cover, and the last page contained no Chinese words. The 12th page was structured very differently from the other pages. Therefore, those three pages were not included in the analysis of the eye movement data, and 10 pages were analyzed. Blue circles on the page represented the text and protagonist zones, which did not appear on the actual page. The boundary of the text zones was defined as a visual angle away from the text, and the circular radius was 75 pixels. The boundary of the protagonist zone was defined as a visual angle away from the protagonist, and the zone’s radius was 150 pixels. The text and picture zones did not overlap, and the text was fixed on the right side of each page (see **Figure 1**).

The PPVT (Dunn, 1965) used in this study was translated and revised by the Shanghai Institute of Pediatrics, and its test–retest reliability was 0.95. Its correlation coefficient with the Wechsler Preschool and Primary Scale of Intelligence (WPPSI) was 0.53 (Gong and Guo, 1984).

The sound used in the reading-with-the-narrator condition was recorded in advance by a non-professional voice dubbing artist majoring in psychology. The recording matched the picture book text. The voice artist read the book vividly, changing her tone, intonation and emotion according to the plot. There was only one sentence on each page (e.g., “A bird comes and takes an apple”). The number of words per sentence was 7–13, and the recording time for each sentence was 2–4 s (*M* = 3, *SD* = 0.67). Children heard the same recording when reading with a narrator.

### Instruments

The digital version of the book was presented on a monitor with a resolution of 1024 pixels × 768 pixels and a refresh rate of 120 Hz. Eye movements were measured with a high-speed eye-tracking device produced by Senso Motoric Instruments (SMI) that had a high sampling rate (250 Hz) and high accuracy (<1). The model was SMI-RED250. A program written in Visual Basic was used to present the stimulus and collect monocular data on the participants. The Python language was used for the eye-gaze analysis.

### Procedure

#### Vocabulary Tests

The PPVT was used to test the children’s receptive vocabulary by asking them to point to one of four pictures that represented a spoken word.

The literacy measure was developed to test the children’s knowledge of the words used in the picture book, and the children were asked to read specific words presented at random. The scores were recorded as 1 (correct) or 0 (incorrect). The correlation coefficient between the children’s PPVT scores and the literacy measure was 0.49 (*p* < 0.01). Detailed information is provided in **Table 1**.

#### Eye Movements

The monitor was positioned approximately 80 cm from the participants’ eyes, and the participants viewed the monitor through a square window to keep their eyes focused on the stimuli. The eye movement measurement process began with a familiarization period. The children were asked to read another picture book to learn how to use the mouse to turn the pages. After ensuring that the children understood the instructions, a standard 9-point calibration procedure was initiated. The participants had to stare directly at the computer screen and look at a series of nine sequential dots. After successful calibration, the experiment began. The children were told that they were going to look at a picture book on the computer. They could turn the pages by clicking the mouse, just like they had learned, until they had read the whole book. During the process, they needed to sit very still and try not to move. After the children were thoroughly prepared, the system randomly selected a reading style for them. The sessions lasted approximately 30 min.

## Results

Each page contained two zones: the protagonist zone and the text zone. The analysis primarily involved three measures. The first measure was the total time spent viewing each zone, which is sensitive to slower and faster cognitive processes (Holmqvist et al., 2011). The second measure was the fixation counts in each zone. Fixation represents the processing of information (Rayner, 1978). When reading materials are attractive or require a high cognitive load, they elicit higher fixation counts and longer viewing times. In this study, fixation was coded for gaze durations of 100 ms or longer. The third measure was time to first fixation (TFF, i.e., the latency from page presentation to the first fixation on the interest zone), which perfectly mirrored previous data on the first fixation (Evans et al., 2009). The shorter the TFF, the more likely the participants’ attention was to be drawn to the target.

### The Influence of Vocabulary and Literacy on the Children’s Reading Behavior

To evaluate the effect of the children’s PPVT and literacy scores on their eye movement variables, a series of simultaneous multiple regression analyses were computed (enter method). The PPVT and literacy scores were entered together as predictor variables, and the eye movement indices (i.e., viewing time, fixation counts, and TFF) were the dependent variables. **Table 2** presents the results of the simultaneous multiple regression analyses. For the TFF of the text zone, the overall regression was significant, *F*(2,51) = 3.22, *p* < 0.05, *R*^2^= 0.13. To further determine the unique contribution of each predictor to the overall regression model, hierarchical regression analyses with the PPVT at step 1 as the control variable and the literacy score at step 2 was performed (enter method). **Table 3** presents the results of the hierarchical regression analyses. The results revealed that the children’s PPVT scores had a significant, unique contribution to the TFF of the text zone and accounted for 12% of the variance, *F*(1,51) = 5.85, *p* < 0.05, *R*^2^= 0.12. After PPVT scores were controlled for, literacy did not explain a significant amount of additional variance (Δ*R*^2^= 0.01, *p* > 0.05). These findings demonstrated that the higher the children’s PPVT scores were, the sooner they fixated on the text. Another noteworthy finding was that the children’s PPVT and literacy scores had a marginal effect on the viewing time for the text zone, *F*(2,51) = 3.04, *p* = 0.058, *R*^2^= 0.08, indicating that the higher the children’s PPVT and literacy scores were, the longer they fixated on the text, to a certain extent. No significant effects between the children’s receptive vocabulary, literacy scores and other measures were found (all *p*s > 0.05).

**Table 2:** Multiple regressions predicting eye movement when reading the picture book.

	Viewing time (ms)				Fixation counts				TFF			
	β	*t*	*R*^2^	*F*	β	*t*	*R*^2^	*F*	β	*t*	*R*^2^	*F*
**Entire book**							
PPVT	0.01	0.06	0	0.04	0.03	0.18	0	0.04	–	–	–	–
Literacy	-0.05	-0.27			0.02	0.1			–	–		
**Protagonist zone**							
PPVT	0.03	0.20	0.02	0.49	0.08	0.44	0	0.13	0.14	0.78	0.02	0.35
Literacy	-0.16	-0.94			-0.08	-0.44			-0.02	-0.13		
**Text zones**							
PPVT	0.18	1.08	0.12	3.04 (marginal significance)	0.17	1.02	0.09	2.09	-0.28	-1.71	0.13	3.22*^∗^*
Literacy	0.23	1.34			0.17	1.04			-0.13	-0.79		

*PPVT, Peabody Picture Vocabulary Test. ^∗^p < 0.05*.

**Table 3:** Hierarchical regression analyses predicting the Time to First Fixation (TFF) on the text zone.

Variable	Step 1		Step 2	
	β	*t*	β	*t*
PPVT score	–0.34	–2.42^∗^	–0.28	–1.71
Literacy level	–	–	–0.13	–0.79
Summary of model	*F* = 5.85^∗^, ∆*F* = 5.85^∗^,		*F* = 3.22^∗^, ∆*F* = 0.63,	
	*R*^2^= 0.12, ∆*R*^2^= 0.12		*R*^2^= 0.13, *∆R*^2^= 0.01	

*PPVT score at Step 1 and literacy level at step 2. ^∗^*p* < 0.05*.

### The Influence of Reading Styles on Children’s Reading Behavior

To investigate the effect of different reading styles on children’s reading behavior, a dependent sample *t*-test was conducted using reading style (independent reading vs. reading with a narrator) as the independent variable and eye movement indices (i.e., viewing time, fixation counts, and TFF) as the dependent variables. The results revealed no significant difference in any of the eye movement indices (all *p*s > 0.05). See details in **Table 4**.

**Table 4:** Means (and standard errors) of eye-gaze indices for each area for different reading styles.

Interest zones	Dependent variable	Independent reading	Reading with a narrator	*t*
		(*N* = 23)	(*N* = 23)	
Entire book	Viewing time	4951.34 (3038.99)	4966.44 (1456.62)	0.02
	Fixation counts	9.10 (6.53)	11.21 (4.21)	1.30
Protagonist zone	Viewing time	1545.30 (802.85)	1700.87 (695.05)	0.70
	Fixation counts	3.26 (1.98)	4.28 (1.92)	1.77
	TFF	822.09 (575.11)	905.60 (395.26)	0.57
Text zones	Viewing time	1141.20 (1288.86)	962.64 (696.35)	–0.59
	Fixation counts	1.90 (3.15)	2.06 (2.02)	0.20
	TFF	1114.31 (783.17)	1360.58 (931.05)	0.97

*The units of means are milliseconds*.

Considering the continuous process of reading, we divided each page into 10 viewing time periods and performed further analysis. We conducted a multilevel analysis using hierarchical linear modeling (HLM) software with time points (TIMEP), reading styles (GROUP), and the PPVT scores as predictor variables; the dependent variable was the probability of fixation on the text zones. The model was as follows:

Level 1:

Y=B0+B1*TIMEP+R

Level 2:

$$\begin{array}{l} \text{B}0=\text{G}00+\text{G}01*\text{PPVT}+\text{G}02*\text{GROUP}\\ \text{B}1=\text{G}10+\text{G}11*\text{PPVT}+\text{G}12*\text{GROUP} \end{array}$$

In the above equation, Y is the index of the dependent variable. B0 is the intercept at Level 1. B1 is the slope, which is the increase in Y for each additional unit of TIMEP. TIMEP is the time point ranging from 1 to 10. GROUP refers to the reading styles, and G00–G12 are the parameters to be estimated. The results of the parameter estimation are presented in **Table 5**.

**Table 5:** A two-level hierarchical regression predicting the children’s fixation probability for the text zone.

	Predictors	Parameter	Estimate	*SE*	*t*	*df*	*p*
B0	INTRCPT2 (Intercept)	G00	-0.05766	0.105507	-0.547	43	0.587
	PPVT	G01	0.001116	0.001184	0.943	43	0.352
	GROUP	G02	0.069851	0.043031	1.623	43	0.112
B1	INTRCPT2 (Slope)	G10	0.00983	0.007059	1.393	454	0.164
	PPVT	G11	0.000048	0.000078	0.607	454	0.544
	GROUP	G12	-0.00703	0.003527	-1.993	454	0.046

**FIGURE 2:**
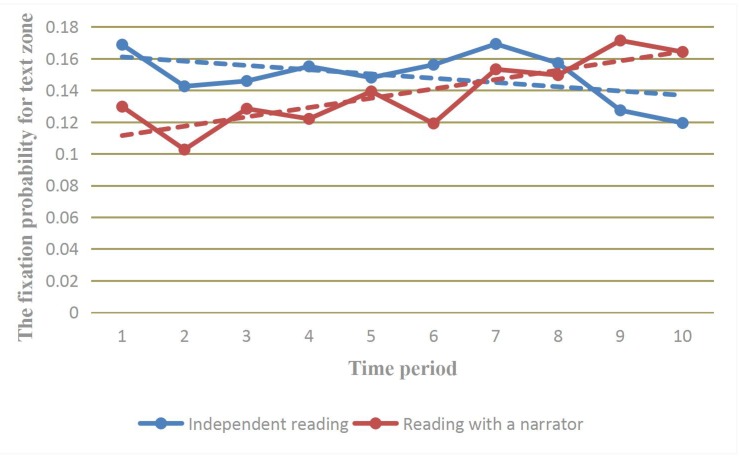
The trend in the change in the children’s fixation probability for the text zone.

According to the HLM analysis, only reading style significantly affected B1 [GROUP (G12 = –0.007, *SE* = 0.0035, *T-ratio* (454) = –1.933, *p* < 0.05)], revealing a significant interaction between time point and reading style; specifically, the rate of change in the fixation probability was significantly lower when the children read independently than when they read with a narrator. Additional information is provided in **Figure 2**. In contrast, there was no significant interaction between time point and PPVT score [G11 = 0.000048, *SE* = 0.000078, *T-ratio* (454) = 0.607, *P* > 0.05], indicating that the children’s receptive vocabulary had no significant effect on the rate of change in the fixation probability.

## Discussion

This study compared the eye movement characteristics of children with different receptive vocabulary and literacy levels when they read independently and when they read with a narrator.

First, we explored the effect of receptive vocabulary and literacy levels on eye movement patterns from a static perspective. In the present study, the children’s vocabulary and literacy levels only influenced the eye movement indices in the text zones. Specifically, the children with higher PPVT scores fixated sooner on the text. The results indicate that children with a larger vocabulary have formed a stronger connection between printed words and their meaning. This finding corroborated the Stroop effect in reading (Roy-Charland et al., 2007). In addition, in the simultaneous multiple regression model, the PPVT and literacy scores had a marginal effect on the viewing time for the text zone. The results indicated that the higher the children’s PPVT and literacy scores were, the longer they fixated on the text to a certain extent. The marginal effect may be related to the small number of subjects, and future studies can increase the number of subjects.

In addition, children with different vocabulary and literacy levels showed no significant difference in terms of total viewing time and fixation counts for the entire page and the protagonist zone. Further analysis revealed that the children spent 33% of their total viewing time on the protagonist zone. The children’s preference for the protagonist may be related to the picture book used in this study and the definition of the interest zones. Specifically, although the picture book included both pictures and text, we did not use the whole picture zone in this study. We defined the protagonist zone as the zone of interest, given that the protagonist appeared on every page, as mentioned earlier. Similarly, in Evans et al. (2009), the same bear appeared on each page of the alphabet book, and the children fixated more quickly and for longer on the picture of the bear than on any of the text zones. These findings suggest that children prefer familiar and repeated features; thus, the method of defining a zone of interest and the repetition of the protagonist may have affected the children’s attention.

Compared with children who are Chinese language speakers, children who know alphabetic languages appear to pay less attention to text. For example, the children in the present study spent 19.38% of their total viewing time on the text zone, which was similar to the 19.9% reported by Han et al. (2011). However, different results were reported by Justice et al. (2005). Their research indicated that children spent only approximately 2.7% of their viewing time on the text. Even in the case of a print-salient storybook, the proportion was only 6%. Of course, variables such as the difference between Chinese and alphabetic languages and the use of different picture books reduce the comparability between research on Chinese subjects and subjects from other countries. Such variables suggest the need for cross-cultural studies.

Second, we explored the effect of reading styles on eye movement patterns. The results revealed no significant difference in the fixation counts and viewing time for protagonist zones for the two reading styles, which was consistent with the results of Han et al. (2011) and the hypothesis, however, in their study, the children spent more time on the text zone when reading independently than when reading with a narrator. In the present study, the children’s viewing time and fixation counts on the text zone did not differ significantly between the two reading styles. Further analysis revealed that the primary difference between the two reading styles was reflected in the slope (the rate of change in the fixation probability with reading time), which was significantly higher for the children who read with a narrator than for those who read independently. Combined with the information presented in **Figure 2**, it is clear that the fixation probability for the text zone decreased with time when the children read independently but increased with time when they read with a narrator.

More precisely, when children read with a narrator, listening to an adult might provide a scaffold for exploring the story by allowing the children to directly match the sound with the pictures (Evans and Saint-Aubin, 2005; Martin-Chang and Gould, 2012), which leads children to pay a great deal of attention to pictures when they begin to read each page. It might be worth noting that the picture book used in this study was relatively simple, and each page included only one short sentence. Consequently, it was very easy for the children to remember the audio content. With the passage of time and the end of the recording, the children’s interest in the text gradually increased. It is possible that they gradually matched the text with the pictures and the sound in their memory to understand the story. Matching sound with text is considered conducive to the formation of a link between speech and text in children (Sulzby, 1985). Although it is not enough to be a mature reader, children in senior kindergarten may have mastered some vocabulary and reading skills. In addition, textual information helps children quickly grasp a story. Therefore, when they need to read independently, they still strive to match text with pictures in order to promote their understanding (Nikolajeva and Scott, 2006). Consequently, it was unsurprising that the probability of fixation on the text decreased with time when the children read independently but increased with time when they read with a narrator and that the total viewing time did not differ significantly between the two reading styles.

In the present study, we simulated an electronic reading situation for children and defined two types of reading styles. Given the abovementioned findings, it is logical to speculate that when children read an e-book with a narrator, the sound might provide a scaffold that allows pre-readers to better understand the story. In addition, listening to an adult and matching text with sound is thought to help children establish and consolidate the links between speech and text (Sulzby, 1985). Considerable research has shown that establishing links between print and speech is a crucial step in learning to read (Blomert, 2011; Jost et al., 2013). A failure to establish an effective link between print and speech can lead to reading disorders (Blomert, 2011). For that reason, when children read with an adult, they are gradually encouraged to attach importance to textual information. Attention to print is the first step in children’s internalization of the forms and functions of print and a key process in forming a speech sound-print connection (Evans and Saint-Aubin, 2005; Justice et al., 2008). Children who have a high level of reading skill and have already established an adequate link between print and sound are able to obtain information quickly by reading text directly, and reading with a narrator does not seem necessary.

### Limitations and Directions for Future Research

This study only examined children’s eye movement patterns during picture book reading. It did not consider reading comprehension or its relationship with eye movements. Future studies should explore the influence of picture book reading on emotions and behavior and/or investigate participants’ understanding and memory level after reading a picture book. Second, the structure of the picture book used in this study was relatively simple. In future studies, more complex picture books should be used to investigate children’s eye movement patterns when reading. Third, e-books often include numerous media effects (such as music) and functions (such as a dictionary). Thus, the effects of e-reader use on children’s language development should be further investigated. Finally, we analyzed the data by subdividing the entire reading time into smaller time periods. This approach was not fully developed, and previous studies offer no precedent in this regard. Our study represents an exploratory attempt, and future studies can improve on our design.

## Conclusion

From a static perspective, children with high receptive vocabulary scores and literacy levels more quickly fixated on the text zones and tended to fixate longer. Based on the dynamic process of reading a picture book, the rate of change in the children’s fixation probability was higher when reading with a narrator than when reading independently. Overall, the fixation probability for the text decreased with time when the children read independently but increased with time when they read with a narrator. For children in senior kindergarten, reading with a narrator may help to establish and consolidate the links between speech and text and thus promote reading acquisition.

## Ethics Statement

This study was conducted in accordance with the ethical standards of the Ethics Committee of the College of Education, Capital Normal University, with written, informed consent from all subjects. Institutional review board approval was obtained for this study. Given that the subjects in this study were children, we received all of the written informed consent forms from their parents. All procedures performed in the study involving human participants were in accordance with the ethical standards of the institutional and national research committee and with the 1964 Helsinki Declaration and its later amendments or comparable ethical standards.

## Author Contributions

YW and YS designed the experiments. YS and ZS collected the data. JD and ZS analyzed the data. ZS and YS wrote the manuscript. ZS and ZM re-edited the manuscript according to the reviewers and the editors’ comments.

## Conflict of Interest Statement

The authors declare that the research was conducted in the absence of any commercial or financial relationships that could be construed as a potential conflict of interest.
